# Identification of thermostability-enhancing mutations in H9N2 avian influenza virus hemagglutinin

**DOI:** 10.1128/jvi.00168-26

**Published:** 2026-03-16

**Authors:** Binjian Liu, Hai Yu, Zhanfei Yan, Shuping​ Zou, Jinyue Guo, Shan Cai, Yingqin Hu, Yu Yang, Yulin Yan, Hao Liu, Kun Mei, Zhili Li, Limei Qin, Yong Li, Shujian Huang, Feng Wen

**Affiliations:** 1 College of Animal Science and Technology, Foshan University, Foshan, Guangdong, China; 2 Shanghai Veterinary Research Institute, Chinese Academy of Agricultural Sciences 118161, Shanghai, China; 3 College of Animal Science and Technology, Jiangxi Agricultural University 91595, Nanchang, Jiangxi, China; University Medical Center Freiburg, Freiburg, Germany

**Keywords:** avian influenza virus, hemagglutinin, H9N2 subtype, HA mutations, protein stability

## Abstract

H9N2 avian influenza viruses (AIVs) remain a significant economic burden on poultry production and a persistent zoonotic threat. Hemagglutinin (HA), a surface glycoprotein mediating viral entry and pathogenesis, critically depends on thermostability for its function. Our previous study indicated that recent H9N2 AIVs have experienced a reduction in hemagglutination activity and exhibit low HA thermostability; however, the underlying molecular determinants for this instability remain poorly defined. To address this gap, we employed an *in vitro*-directed evolution approach to identify HA mutations that enhance thermostability. By subjecting a diverse HA mutant library to iterative heat selection at 48°C, we isolated several HA-stabilizing mutations, including L29I, N133S, N210D, G266R, D387N, A423T, and E509G, and confirmed their effect by site-directed mutagenesis. Further characterization revealed a complex interplay between HA stability, receptor binding specificity, and acid tolerance. Our findings demonstrate that enhancing HA stability can exert pleiotropic effects on key viral properties, highlighting the importance of understanding these relationships for developing effective mitigation strategies against H9N2 AIVs.

**IMPORTANCE:**

H9N2 avian influenza viruses pose a persistent threat to poultry production and human health, demanding improved control strategies. This study addresses a key knowledge gap by uncovering the molecular determinants that modulate the stability of the hemagglutinin (HA) protein in H9N2 viruses. We identify specific HA mutations that increase thermostability, a property potentially linked to environmental persistence. Furthermore, our findings demonstrate a critical interplay between HA stability and essential viral functions, including receptor binding, hemagglutination activity, replication kinetics, and overall acid tolerance. By disentangling these properties, we provide insights into the mechanisms underlying HA-mediated viral entry and infectivity, which could inform the development of more effective vaccines and therapeutics.

## INTRODUCTION

H9N2 avian influenza viruses (AIVs) represent a persistent threat to global poultry production, causing substantial economic losses while simultaneously posing a potential risk to human health due to their documented capacity for cross-species transmission. H9N2 AIVs have been shown to infect a diverse range of mammalian hosts, including swine, canines, equines, bats, mink, pika, and humans (1–9), underscoring their broad host tropism and adaptive potential. Although human infections with H9N2 viruses remain infrequent, and sustained human-to-human transmission has not been observed, experimental studies in the ferret model have demonstrated airborne transmission of H9N2 AIVs (10–12), highlighting the need for continued surveillance and risk assessment, given their demonstrated propensity for interspecies spread.

As a principal surface glycoprotein of AIVs, hemagglutinin (HA) serves as a critical determinant of viral host range, transmissibility, and pandemic potential in humans, properties intimately linked to its receptor-binding avidity and overall stability (13–17). The HA protein, cleaved into HA1 and HA2 subunits (18), orchestrates viral entry: HA1 mediates attachment to host cells via sialic acid receptors, dictating host tropism, while HA2 undergoes acid-catalyzed conformational rearrangements that trigger fusion of the viral and host cell membranes, initiating infection (19–21). The HA stability is a key virological trait that enhances mammalian adaptation of influenza viruses by enabling precise control of membrane fusion, thereby promoting airborne transmission and highlighting its function as a selective bottleneck during cross-species spread (22–25).

H9N2 AIVs exhibit widespread circulation in poultry and wild bird reservoirs across Asia, the Middle East, North Africa, and West Africa (26–28), underscoring their broad geographic distribution and potential for zoonotic emergence. Given the HA protein’s pivotal role in viral replication, stability, and transmission (25, 29–31), its biophysical properties are critical determinants of viral fitness, making HA stability of particular relevance. Indeed, engineered H5N1 viruses bearing human-type receptor-binding mutations in HA were unable to achieve airborne transmission in ferrets; however, acquisition of compensatory mutations, such as T318I and H110Y, which augment HA thermostability and lower the pH threshold for activation, restored airborne transmissibility (15, 16, 32). Elevated temperatures may promote HA conformational changes, facilitating the transition from a non-fusion to a fusion-competent state even at near-neutral pH, thereby obviating the need for stringent acidic activation (18, 25). Furthermore, the thermostability of HA holds significant implications for influenza vaccine development. Engineering enhanced thermostability into H1N1 and H3N2 vaccine strains could yield more robust and efficacious immunogens, which would exhibit improved stability under diverse storage and deployment conditions (24, 33–35). Indeed, HA thermostability is critically linked to AIV pathogenesis and cross-species transmission, as it dictates viral persistence in the environment and within new hosts (24, 36–38).

Despite the recognized importance of HA stability in AIV, the HA mutations governing H9N2 virus thermostability remain unclear. Here, we identify a novel panel of HA mutations that confer significantly enhanced thermostability, providing both mechanistic insights and a foundation for improved H9N2 vaccine design.

## MATERIALS AND METHODS

### Cells and viruses

Madin-Darby canine kidney (MDCK, CCL-34), African green monkey kidney (Vero, CCL-81), and Human Embryonic Kidney 293T (HEK293T, CRL-3216) cells were obtained from the American Type Culture Collection (ATCC). Cells were cultured in Dulbecco’s modified Eagle’s medium (DMEM; Gibco, USA) supplemented with 10% fetal bovine serum (FBS; Gibco) and penicillin/streptomycin (100 U/mL and 100 μg/mL, respectively; Thermo Fisher Scientific, USA) at 37°C in a 5% CO_2_ atmosphere. Wild-type (WT) A/chicken/Baise/0701/2019(H9N2) (BS19) virus (GenBank: OR398789 to OR398796), which lost hemagglutination ability at 37°C (39), along with A/Puerto Rico/8/1934 (H1N1) (PR8) and A/California/04/2009(H1N1)(CA/04) viruses, were propagated in 10-day-old specific pathogen-free (SPF) chicken eggs (Xinxing Dahuanong Egg Co., LTD, China) at 37°C for 72 h. Viral stocks were stored at −80°C. Viral titers were determined by 50% tissue culture infectious dose (TCID_50_) assay in MDCK cells.

### Construction of mutant plasmid libraries

Mutant libraries were generated using a combination of error-prone PCR (epPCR) and site-directed mutagenesis, as described previously (40). Briefly, the ectodomain (residues 19−530) of the BS/19 HA protein was divided into four overlapping regions of approximately 128 residues each, and region-specific primers were designed for random mutagenesis: L1-F (5′-GCAGCAACAGTAAGCAATGCA-3′), L1-R (5′-ACCTGAGCATGTGTTGCTTGT-3′), L2-F (5′-TGGAATGTGTCTTACGATGGG-3′), L2-R (5′-TAGATCAGTCCTCAGAATTCT-3′), L3-F (5′-CTTTCAGGAGAGAGCCACGGA-3′), L3-R (5′-ACTGAATTCATGGTCAATGAT-3′), L4-F (5′-AAAATGAACAAGCAATATGAA-3′), and L4-R (5′-AATCACAAGAGATGAGGCGAC-3′). Random mutations were introduced into each region using the QuickMutation Random Mutagenesis Kit (Beyotime, China) following the manufacturer’s protocol. PCR products were purified using the GeneJET Gel Extraction Kit (Thermo Fisher Scientific).

These purified, randomly mutagenized PCR products (125 ng each) served as primers for site-directed mutagenesis using the Quick Mutation Gene Site-Directed Mutagenesis Kit (Beyotime) as described (41). Following *Dpn*I digestion (Thermo Fisher Scientific) at 37°C for 1 h, the products were transformed into XL1-Blue Supercompetent Cells (Agilent, USA). Plasmid DNA was extracted from bacterial colonies using the GeneJET Plasmid Miniprep Kit (Thermo Fisher Scientific). We performed Sanger sequencing on a representative pool of 20 plasmid clones isolated from the XL1-Blue transformation to verify the maintenance of a random library during amplification. This confirmed mutation rates of 1−3 amino acids per region, as previously achieved through epPCR optimization.

### Virus rescue

Mutant viruses were rescued using a plasmid-based reverse genetics system as described previously (42). Briefly, for each of the four HA mutant library constructions, a mixture of plasmids was co-transfected into a co-culture of 293T and MDCK cells (20:1 ratio) using Lipo6000 Transfection Reagent (Beyotime) according to the manufacturer’s instructions. Crucially, trypsin was not included in the culture medium during this initial transfection and virus rescue step. The plasmid mixture comprised 0.25 μg of each HA mutant plasmid library, the NA plasmid from the BS19 virus, and the six internal gene plasmids (PB2, PB1, PA, NP, M, and NS) from the PR8 virus. Each mutant library was generated from multiple independent transfections to ensure genetic diversity. Two hours post-transfection, the transfection medium was replaced with Opti-MEM (Gibco) containing N-tosyl-L-phenylalanine chloromethyl ketone (TPCK)-treated trypsin (1 μg/ml; Sigma-Aldrich, USA). After 48 h of transfection, pooled supernatants from each library were collected and amplified in MDCK cells supplemented with TPCK-treated trypsin (1 μg/mL) to generate the respective HA mutant viral stocks.

Following amplification, the resulting viral supernatants were titrated using both a HA assay and a TCID_50_ assay. The HA assay quantifies viral particle quantity based on hemagglutinin binding, while the TCID_50_ assay measures infectivity in cell culture. Employing both assays allows us to assess the association between particle number and infectivity and to investigate if mutations affecting thermostability disproportionately impact functional HA integrity versus overall replication capacity, thereby providing a more comprehensive characterization of viral stocks.

### HA assay and TCID_50_ assay

HA assays were performed using 0.5% chicken red blood cells (CRBCs) following a standard protocol as described previously (43). The HA titer was determined as the reciprocal of the highest dilution at which complete hemagglutination of 0.5% CRBCs was observed. Viral infectivity titers were determined using the TCID_50_ assay. Briefly, viruses were serially diluted 10-fold in Opti-MEM (Gibco) containing TPCK-treated trypsin (1 μg/mL) and used to infect MDCK cell monolayers cultured in 96-well plates. MDCK cells were cultured for 72 h at 37°C in a 5% CO_2_ incubator. Subsequently, 50 µL of supernatant was mixed with an equal volume of 0.5% CRBCs and incubated at room temperature (RT) for 30 min to assess the cumulative number of positive units. The TCID_50_, with a detection limit of approximately 10^1.5^ TCID_50_/mL, was calculated using the Reed-Muench method (44).

### Site-directed mutagenesis

Site-directed mutagenesis was performed to validate the thermostability-enhancing mutations identified from the random library. Mutations were introduced into the HA gene using the Quick Mutation Gene Site-Directed Mutagenesis Kit (Beyotime), following the manufacturer’s instructions and a referenced protocol. The primer pairs used for each mutation were as follows: for L29I, forward 5′-CACAGAAACTGTGGACACAATAACAGAAAACAATGTCCC-3′ and reverse 5′-GGGACATTGTTTTCTGTTATTGTGTCCACAGTTTCTGTG-3′; for A118V, forward 5′-GATCACTTTTTAGTTCTGTTAGGTCATATCAAAGAATC-3′ and reverse 5′-GATTCTTTGATATGACCTAACAGAACTAAAAAGTGATC-3′; for N133S, forward 5′-CAGACACAATTTGGAGTGTGTCTTACGATGG-3′ and reverse 5′-CCATCGTAAGACACACTCCAAATTGTGTCTG-3′; for N210D, forward 5′-GCAACGGGAGAAATAGATAGGATCTTCAAACC-3′ and reverse 5′-GGTTTGAAGATCCTATCTATTTCTCCCGTTGC-3′; for G266R, forward 5′-CAGGAGAGAGCCACAGAAGAATTCTGAGG-3′ and reverse 5′-CCTCAGAATTCTTCTGTGGCTCTCTCCTG-3′; for D387N, forward 5′-GTAAATAATATAGTCAACAAAATGAACAAG-3′ and reverse 5′-CTTGTTCATTTTGTTGACTATATTATTTAC-3′; for A423T, forward 5′-CCAGGATATATGGACATATAATGCAG-3′ and reverse 5′-CTGCATTATATGTCCATATATCCTGG-3′; and for E509G, forward 5′-GGGTCAAGCTGGGATCTGAAGGAAC-3′ and reverse 5′-GTTCCTTCAGATCCCAGCTTGACCC-3′. All mutant HA constructs were verified by Sanger sequencing. Subsequently, reassortant H9N2 viruses, each carrying one of the above HA mutations on a background of internal genes from the PR8 virus, were rescued using a plasmid-based reverse genetics system.

### Replication kinetics

A standard *in vitro* replication curve was generated, following established protocols (45), to assess the impact of identified HA mutations on viral replication. MDCK cells were infected with viruses at a multiplicity of infection (MOI) of 0.01. After a 1 h adsorption period at 37°C, the cell monolayer was washed twice with phosphate-buffered saline (PBS) to remove unbound virus particles. The cells were then overlaid with infection medium consisting of Opti-MEM (Gibco), supplemented with 1 μg/mL TPCK-treated trypsin and a penicillin (100 U/mL)-streptomycin (100 μg/mL) antibiotic solution. Culture supernatants were collected at designated time points post-infection (12, 24, 36, 48, 60, and 72 h), aliquoted, and stored at −80°C. The collected samples were subsequently titrated to determine viral infectivity using the TCID_50_ assay on MDCK cells.

### HA thermostability assay

The HA thermostability assay was performed as described by Hanson et al. with minor modifications (46). Briefly, aliquots of virus or mutant libraries were transferred to 0.2 mL PCR tubes and incubated in a heating block at 48°C for 10 min, 20 min, 30 min, 1 h, or 2 h. Following heat treatment, samples were immediately placed on ice. Infectivity and HA titers were then determined using the TCID_50_ and HA assays, respectively.

### Plaque assay

Following incubation at various temperatures, virus libraries were titrated by plaque assay with a limit of detection ranging from 10 to 50 PFU/mL of the stock virus, as described previously (46). Briefly, viruses were serially diluted 10-fold and used to inoculate confluent MDCK cells in 6-well plates. After a 2-h adsorption period, the cells were washed twice with PBS and overlaid with Eagle’s minimal essential medium (MEM) containing 0.3% bovine serum albumin (BSA), 1% agarose, and TPCK-treated trypsin (1 μg/mL; Sigma-Aldrich, USA). The plates were incubated at 37°C with 5% CO_2_ for 72 h, after which plaques were randomly isolated and amplified in MDCK cells.

### Erythrocyte elution assay

To investigate the effect of HA protein mutations identified from a thermostability library, erythrocyte elution assays were performed to determine the HA elution kinetics of H9N2 viruses. Reassortant viruses rg-L29I, N133S, N210D, G266R, D387N, A423T/E509G, and WT virus were used in this assay, with minor modifications to a previously described protocol (47). Briefly, viruses were adsorbed onto a 0.5% suspension of CRBCs at 4°C for 30 min. Elution of viruses from erythrocytes was then monitored at RT. HA titers were determined hourly for 8 h. The percentage of HA titer at each time point relative to the initial HA titer at 4°C was calculated. Experiments were performed in triplicate.

### Acid stability assay

To assess the acid stability of the viruses, the pH of phosphate-buffered saline (PBS) was adjusted to a range of values (pH 7.0, 6.5, 6.0, 5.5, 5.0, and 4.5) using citric acid to mimic the pH conditions encountered within endosomes during viral entry. Test viruses were diluted to a concentration of 10^6^ TCID_50_ per 100 μL in PBS at each of the indicated pH values. The resulting virus mixtures were then incubated at 37°C for 1 h to simulate the conditions within cellular endosomes. Following incubation, the TCID_50_ of each sample was determined using MDCK cells to quantify the remaining infectious virus. All assays were performed in triplicate for each pH condition to ensure reproducibility and statistical validity.

### Receptor-binding specificity assay

The binding specificity of H9N2 mutants to sialylated receptor analogs was determined using a solid-phase binding assay as described previously (41). The receptor analogs used were biotinylated sialylglycopolymers, specifically Neu5Acα2-3Galβ1-4GlcNAcβ-sp3-PAA-biot (3′SLN-BP) and Neu5Acα2-6Galβ1-4GlcNAcβ-sp3-PAA-biot (6′SLN-BP) (Sigma-Aldrich). These glycans were serially diluted 2-fold and added to Streptavidin Matrix Coated 96-Well Plates (BEAVER, China), followed by incubation at 4°C for 12 h. Three independent binding experiments were performed for each glycan concentration. Subsequently, the 96-well plates were blocked with 5% bovine serum albumin (BSA; Sigma-Aldrich) at 4°C for 12 h to prevent non-specific binding. After blocking, the plates were incubated with virus solutions (16 HA units/50 µL per well) at 4°C for 2 h. Following virus adsorption, the plates were washed extensively with PBS to remove unbound virus. Viral RNA was then extracted after incubating the plates with lysis buffer at 55°C for 10 min. Viral RNA copies were quantified using one-step real-time quantitative reverse transcription PCR (qRT-PCR) as described previously (41).

### Statistical analyses

Statistical analyses were conducted using GraphPad Prism 8 software (GraphPad Software Inc., San Diego, CA, USA). The data are shown as the mean ± SD for all assays. For comparisons between two groups, a two-tailed unpaired Student’s *t*-test was used. For experiments involving multiple groups, statistical significance was determined using two-way analysis of variance (ANOVA), followed by Dunnett’s multiple comparisons test. The levels of statistical significance were defined as follows: *P* < 0.05 (*), *P* < 0.01 (**), *P* < 0.001 (***), and *P* < 0.0001 (****), with "ns" indicating no significant difference.

## RESULTS

### Generation of HA mutant libraries based on the epPCR mutagenesis strategy

Our previous studies revealed a marked reduction in HA activity and thermostability of the H9N2 AIV following a 40-min incubation at 45°C (41). To identify key residues influencing HA stability, we sought to isolate novel mutations within the BS19 HA protein that enhanced thermostability. We previously observed that a single amino acid substitution, N141K, in the HA protein of BS19 conferred enhanced thermostability (41).

**Fig 1:**
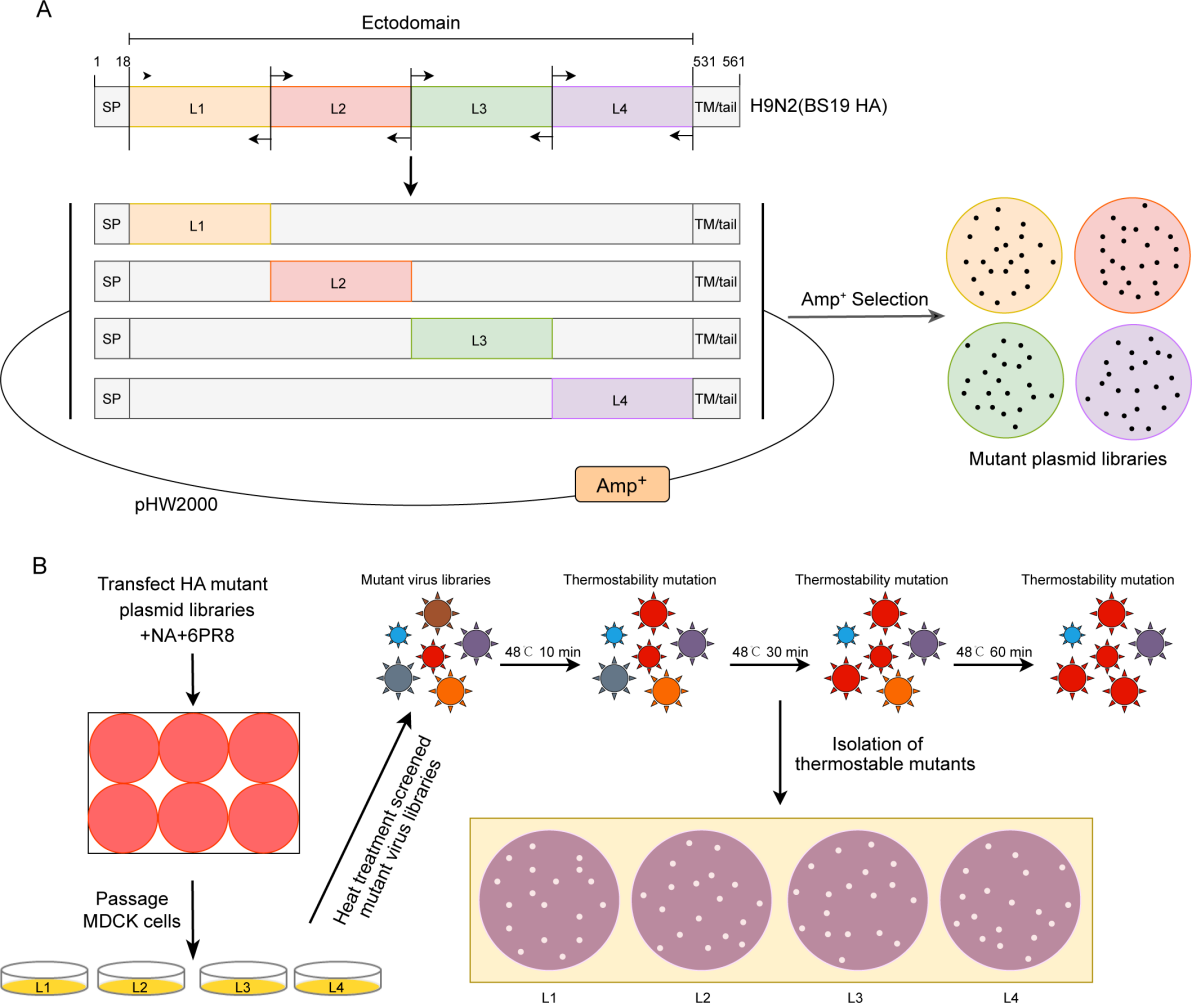
Schematic representation of the screening and identification strategy for H9N2 HA-stabilizing mutant viruses. (**A**) Four target regions (L1−L4) spanning the HA ectodomain (amino acids 19−530) were subjected to random mutagenesis using error-prone PCR (epPCR). The wild-type (WT) HA protein includes the signal peptide (SP; amino acids 1−18) and the transmembrane/cytoplasmic tail region (TM/tail; amino acids 531−560). Mutated sequences were then introduced into the plasmid backbone via site-directed mutagenesis, generating mutant plasmid libraries. To confirm the presence of approximately 1−4 amino acid mutations within each region, clones were randomly selected and sequenced. (**B**) The HA mutant plasmid libraries, along with plasmids encoding BS19 NA and six internal gene segments from PR8, were co-transfected into co-cultures of 293T and MDCK cells for virus rescue. The resulting mutant virus libraries were then subjected to three rounds of heat treatment at 48°C (10 min, 30 min, and 60 min) to enrich for thermostable mutants. Thermostable HA mutants were subsequently isolated via plaque assay.

To systematically screen for novel amino acid substitutions within the BS19 HA ectodomain that could improve its thermostability, we employed an error-prone PCR (epPCR)-based mutagenesis strategy to introduce random mutations throughout the HA-coding region. The full ectodomain sequence of the BS19 HA protein (amino acids 19−530) was divided into four overlapping regions, each approximately 128 amino acids in length. These regions, designated Libraries 1 (L1), 2 (L2), 3 (L3), and 4 (L4), were designed to comprehensively cover the HA ectodomain (Fig. 1).

Each HA fragment was subjected to epPCR using specifically designed primers (see Materials and Methods for details). To achieve a controlled and manageable mutation rate, epPCR cycle parameters were carefully optimized. Following optimization, Sanger sequencing of randomly selected clones from each library confirmed a mutation frequency of 1−3 amino acid substitutions per HA fragment. This mutation rate was considered optimal for generating a diverse library of HA variants while minimizing the introduction of detrimental mutations that would disrupt HA function.

The epPCR products, containing the introduced random mutations, were used as megaprimers in a site-directed mutagenesis approach to replace the WT HA sequences in the virus rescue plasmid vector. Plasmid DNA was extracted from the resulting bacterial colonies, creating four distinct plasmid libraries, each harboring a diverse collection of HA variants with extensive random mutations. To enable rapid assessment of these HA variants, we used a reverse genetics system with a PR8 genetic background to rescue and amplify the mutant viruses. This approach allowed us to introduce the mutated HA genes into a well-characterized and readily replicable virus. The plasmid libraries, along with the N2 and remaining six PR8 genes, were co-transfected into 293T cells to generate reassortant viruses. The supernatant from the transfected 293T cells, containing the rescued viruses, was harvested and amplified in MDCK cells, resulting in four mutant virus libraries (L1, L2, L3, and L4) corresponding to the initial HA regions. As a control, we also rescued the WT virus with internal genes from PR8, designated rg-WT (Fig. 1).

### Screening for HA mutants with enhanced thermostability

To isolate HA mutants exhibiting enhanced thermostability, we subjected mutant virus libraries to sequential heat treatments, leveraging the principle that viruses with lower physical stability are selectively inactivated by controlled heat exposure (Fig. 1). Heat treatment at neutral pH can induce conformational changes in the HA protein that mimic those triggered by low-pH conditions within late endosomes, potentially selecting for mutants with improved stability during virus entry.

**Fig 2:**
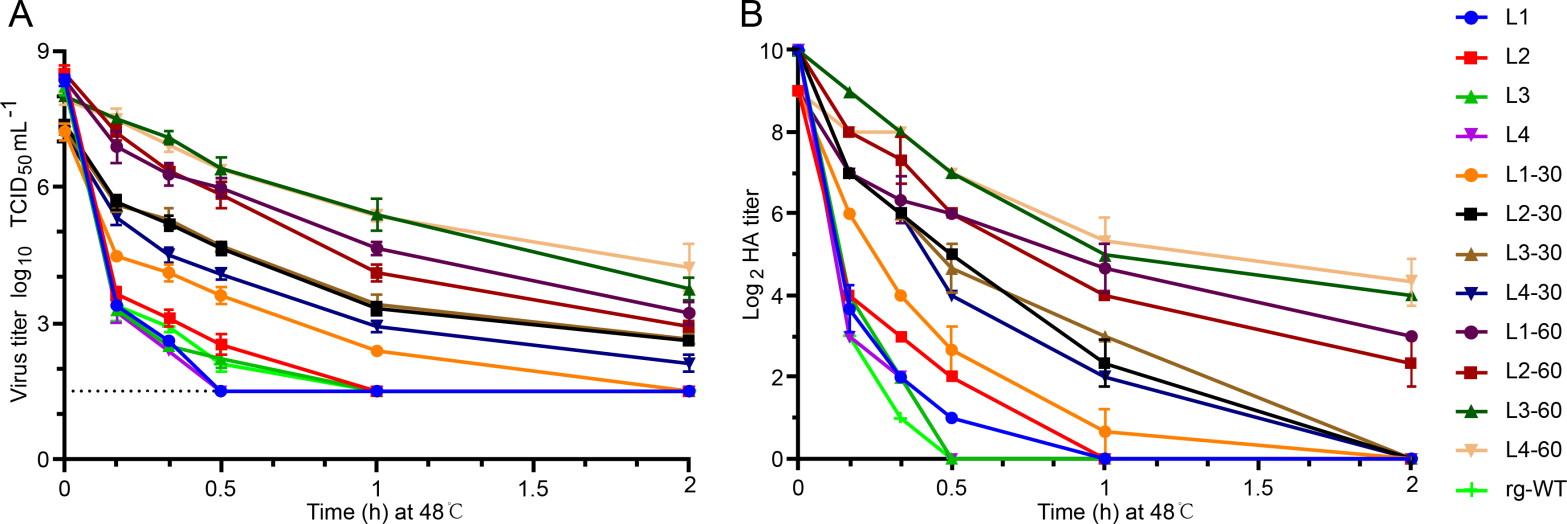
Selection of H9N2 HA-stabilizing mutant viruses. The thermostability of four HA mutant virus libraries, derived from the BS19/PR8 virus, was assessed after three consecutive rounds of heat treatment at 48°C. Infectivity titers (**A**) and hemagglutination (HA) titers (**B**) of the virus libraries were determined using TCID50 and HA assays, respectively, at the following time points: 0, 10 min, 20 min, 30 min (0.5 h), 60 min (1 h), and 120 min (2 h). Data are presented as mean ± standard deviation (SD) for each time point. The dashed line indicates the limit of detection (LOD) of the titration assay, which was 10^1.5^ TCID_50_/mL.

We first assessed the inactivation kinetics of the four mutant virus libraries (L1, L2, L3, and L4) to determine optimal conditions for selective inactivation. Incubation of each library at 48°C for 10 min resulted in a substantial reduction in infectivity titer (approximately five log10 units) (Fig. 2A) and a 32-fold to 128-fold decrease in HA titer (Fig. 2B). A similar heat treatment of the rg-WT virus also resulted in an approximate five log10 unit reduction in infectivity titer, but the HA titer decreased by only 64-fold, suggesting slightly higher thermostability of rg-WT. These results indicated that 48°C for 10 min was a suitable starting point for selecting thermostable mutants (Fig. 2).

Subsequently, we performed three rounds of heat treatment to enrich for thermostable mutants. The mutant virus libraries were incubated at 48°C for 10 min (L1−10, L2−10, L3−10, and L4−10), 30 min (L1−30, L2−30, L3−30, and L4−30), and 60 min (L1−60, L2−60, L3−60, and L4−60), with amplification in MDCK cells after each round. After the third heat treatment, the four mutant virus libraries (L1−60, L2−60, L3−60, and L4−60) exhibited significantly enhanced thermostability compared to the original libraries (*P* < 0.001), as evidenced by increased viral and HA titers following heat exposure. The L3−60 and L4−60 mutant libraries demonstrated higher thermostability than the L1−60 and L2−60 mutant libraries, suggesting that the regions represented by L3 and L4 may contain residues particularly important for HA thermostability and/or be more amenable to stabilizing mutations.

### Isolation and characterization of thermostable HA mutants

**Fig 3:**
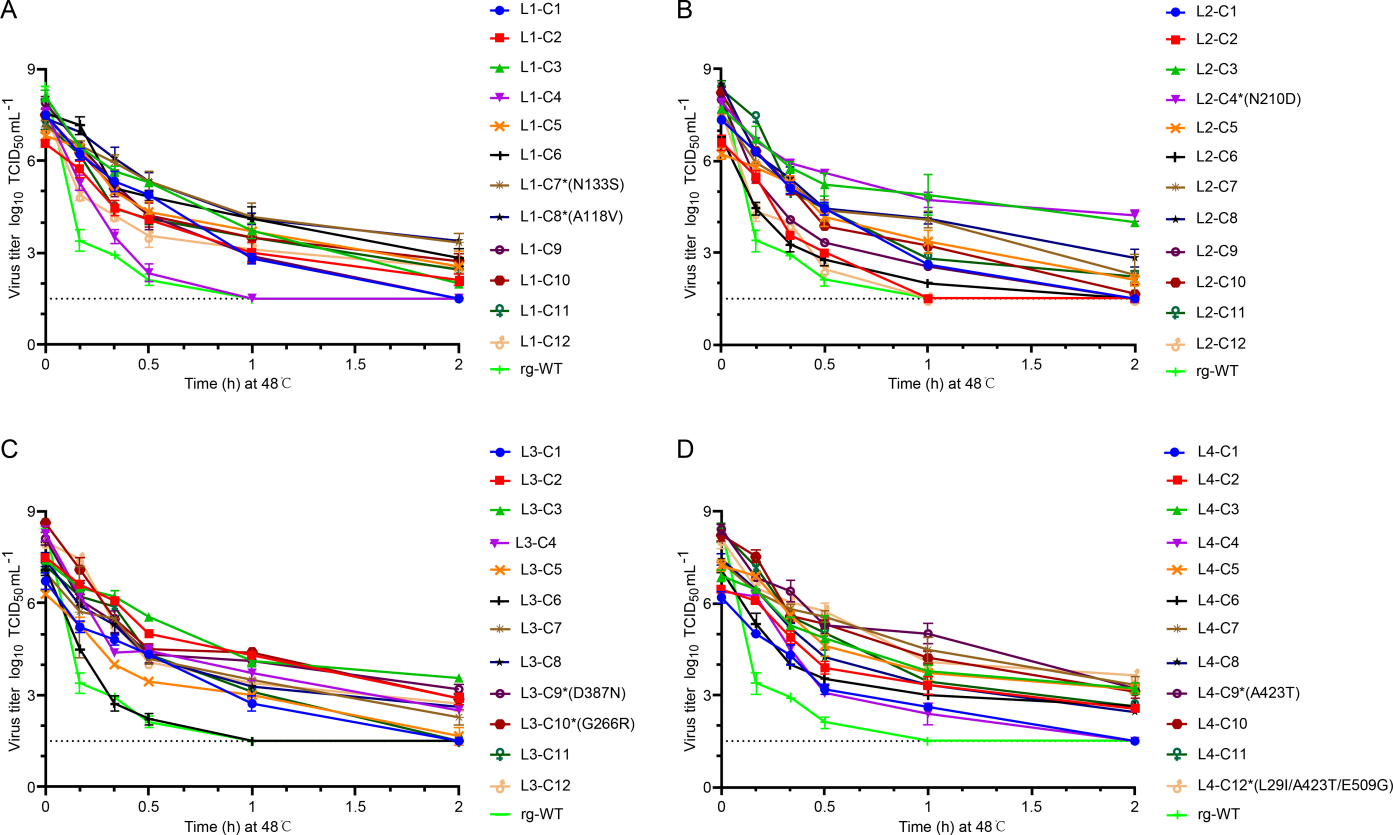
Infectivity titers of selected H9N2 HA-stabilizing mutant viruses. Following the third round of heat treatment and subsequent plaque purification on MDCK cells (performed in triplicate), 12 plaques (C1−C12) were randomly selected from each mutant virus library L1 (**A**), L2 (**B**), L3 (**C**), and L4 (**D**) and amplified in MDCK cells. The infectivity titers of these selected thermostable HA mutant viruses were then measured after incubation at 48°C for the indicated time periods. Viral infectivity was determined using the TCID_50_ assay. The dashed line indicates the limit of detection (LOD) of the titration assay, which was 10^1.5^ TCID_50_/mL.

**Fig 4:**
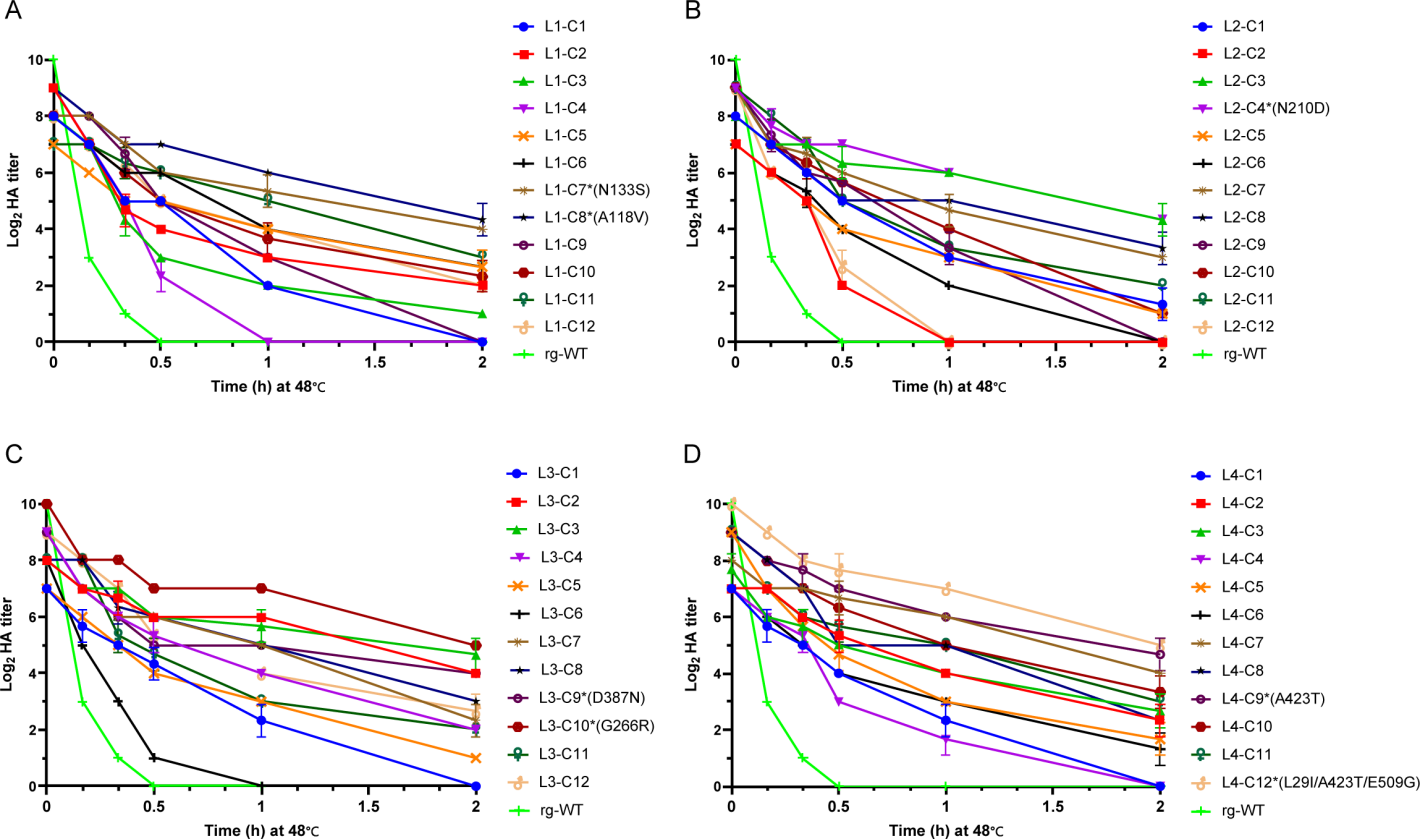
Hemagglutination titers of selected H9N2 HA-stabilizing mutant viruses. Following the third round of heat treatment and subsequent plaque purification on MDCK cells (performed in triplicate), 12 plaques (C1−C12) were randomly selected from each mutant virus library L1 (**A**), L2 (**B**), L3 (**C**), and L4 (**D**) and amplified in MDCK cells. The hemagglutination (HA) titers of these selected thermostable HA mutant viruses were then measured after incubation at 48°C for the indicated time periods. HA titers were determined using a hemagglutination assay.

Following heat selection, the L1−60, L2−60, L3−60, and L4−60 virus libraries, enriched for thermostable variants, were further processed to isolate individual clones. Each library was incubated at 48°C for 1 h to impose an additional selection pressure. The treated libraries were serially diluted and used to inoculate MDCK cells, and individual plaques were isolated via plaque purification. Twelve plaques were randomly selected from each library, resulting in a total of 48 isolates. These isolates were amplified in MDCK cells to generate sufficient virus stock for subsequent characterization. To assess thermostability, infectivity and HA titers of the isolated viruses were determined following incubation at 48°C for various durations. The results revealed substantial variation in thermostability among the 48 isolates (Fig. 3 and 4). Notably, the majority of mutant viruses isolated from the four libraries exhibited higher thermostability at 48°C compared to the rg-WT virus, as demonstrated by higher infectivity (Fig. 3A through D) and HA titers (Fig. 4A through D) across the tested time points.

### Mutations in HA associated with increased thermostability

To investigate the HA mutations contributing to the enhanced thermostability of the selected viruses, we analyzed the HA sequences of the 11 most thermostable viruses, chosen from the four mutant virus libraries. Sanger sequencing revealed several amino acid substitutions in the HA protein that likely underpin the observed increase in stability.

**Fig 5:**
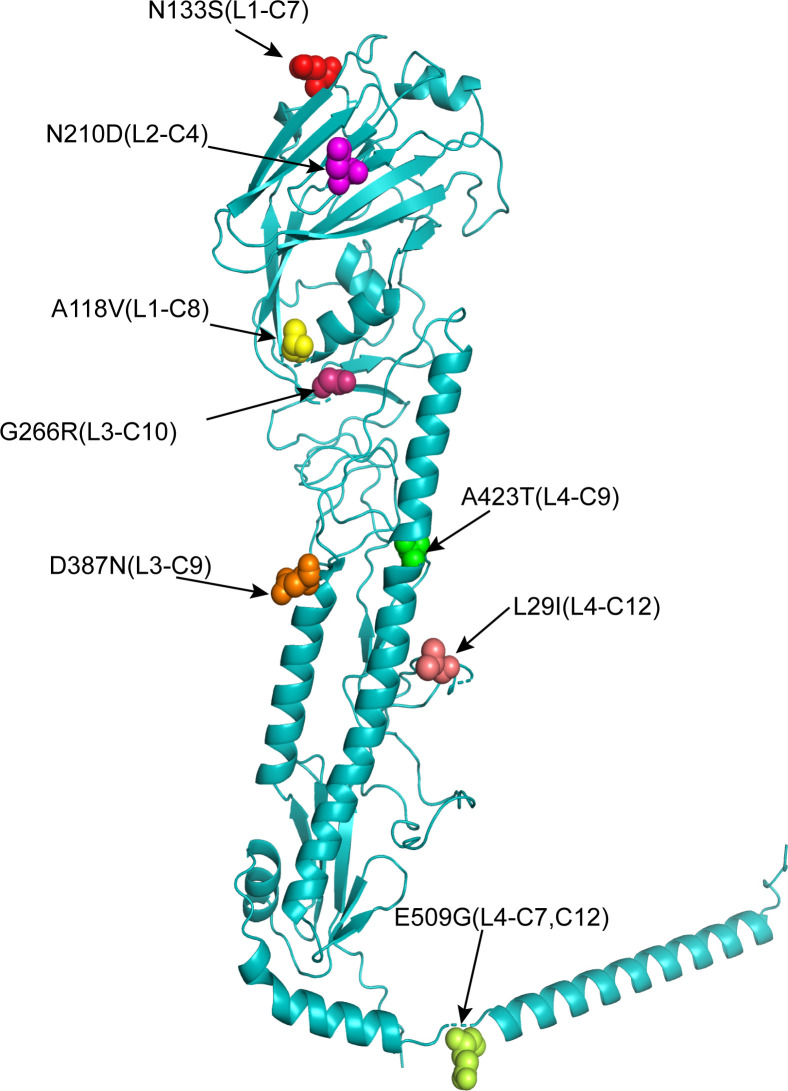
Location of enhanced thermostability mutations on the hemagglutinin (HA) monomer of H9N2 AIV. The monomeric structure of the BS19 virus HA protein is shown as a ribbon diagram, with identified thermostability mutations mapped and represented as colored spheres. The positions of the following mutations are indicated: A118 (yellow), N133 (red), N210 (magenta), G266 (pink), D387 (orange), L29 (light pink), A423 (green), and E509 (light yellow).

The identified mutations were distributed across the HA protein. Specifically, the L1 library contained A118V and N133S (130-loop of RBD); L2 contained N210D (220-loop of RBD); L3 contained G266R and D387N (HA stalk); and L4 contained A423T and L29I/A423T/E509G (HA stalk) (Fig. 5). The L29I mutation in the L4 library, residing at residue 29 (a target of L1 mutagenesis), is hypothesized to represent an adaptive mutation acquired during cell passage. Notably, N210D, D387N, and A423T were the most frequently observed mutations, suggesting they play a key role in enhancing viral thermostability and may act synergistically to further improve HA thermostability.

### Functional validation of HA mutations enhancing viral thermostability

To confirm that the increased viral thermostability was attributable to the identified HA mutations, we introduced each mutation individually into the HA gene of the BS19 virus, rescuing a panel of nine reassortant mutant viruses. Among these, one mutant originating from the L4 library contained a triple mutation (L29I/A423T/E509G). To delineate the individual contribution of each constituent mutation and explore potential synergistic effects, we generated viruses carrying each of the three corresponding single-site mutations (rg-L29I, rg-A423T, and rg-E509G), as well as a double mutant (rg-A423T/E509G). The L29I single mutant was excluded from the synergy analysis due to its likely adaptive role.

**Fig 6:**
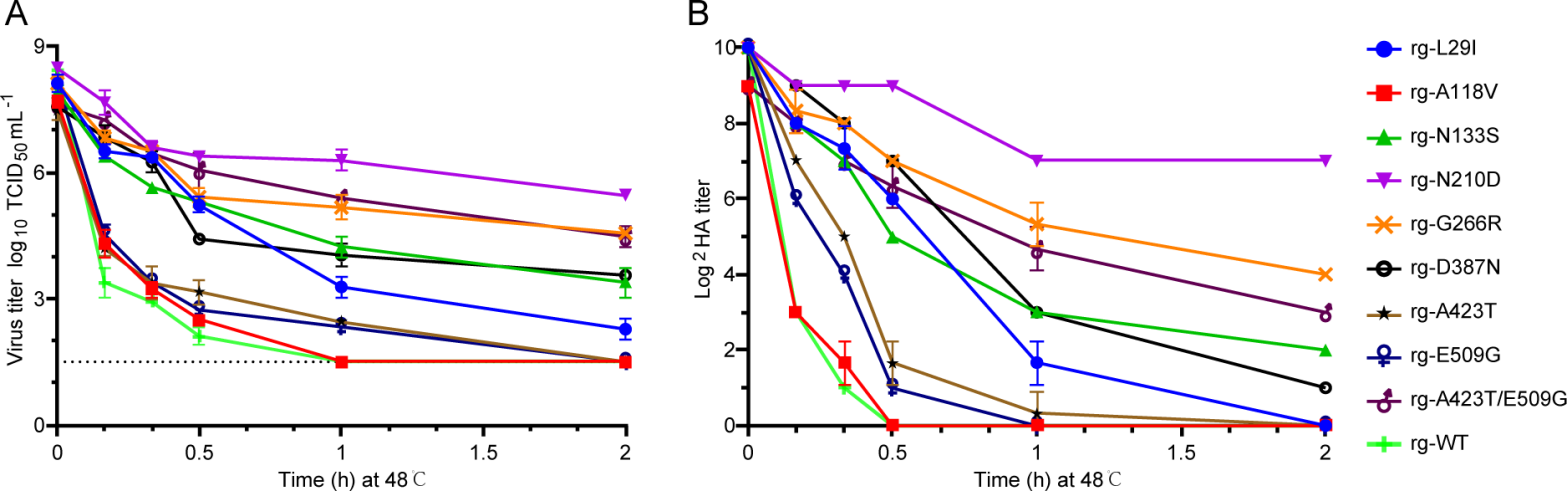
Validation of HA-stabilizing substitutions of H9N2 AIV. The indicated HA substitutions were introduced into the HA gene via site-directed mutagenesis, and 6+2 rgH9N2 mutant viruses, containing internal genes from the PR8 virus, were generated using plasmid-based reverse genetics. Thermostability of the rescued viruses was assessed by measuring (**A**) infectivity titers (log_10_ TCID_50_/mL) and (**B**) hemagglutination (HA) titers (log_2_ HA titer) following incubation at 48°C for the indicated durations. Data for each virus are color-coded as follows: rg-L29I (blue), rg-A118V (red), rg-N133S (green), rg-N210D (purple), rg-G266R (orange), rg-D387N (black), rg-A423T (yellow), rg-E509G (dark blue), rg-A423T/E509G (pink), and rg-WT (light green). The dashed line indicates the limit of detection (LOD) of the titration assay, which was 10^1.5^ TCID_50_/mL.

The rescued viruses—rg-L29I, rg-A118V, rg-N133S, rg-N210D, rg-G266R, rg-D387N, rg-A423T, rg-E509G, and rg-A423T/E509G—were exposed to 48 °C for varying time periods. Viral thermostability was evaluated by measuring residual infectivity (Fig. 6A) and HA activity (Fig. 6B). Both the rg-WT and the rg-A118V mutant rapidly lost infectivity and hemagglutination capacity upon heating, indicating poor thermal stability. In contrast, viruses carrying the N133S, N210D, G266R, or D387N single-point mutations showed significantly enhanced thermostability compared to rg-WT (*P* < 0.001). The A423T and E509G single mutations individually conferred only marginal stability gains; however, when combined in the rg-A423T/E509G double mutant, a substantial improvement in thermostability was observed (*P* < 0.001), suggesting a clear synergistic interaction between these two sites.

### Receptor binding specificity of viruses with HA thermostability mutations

**Fig 7:**
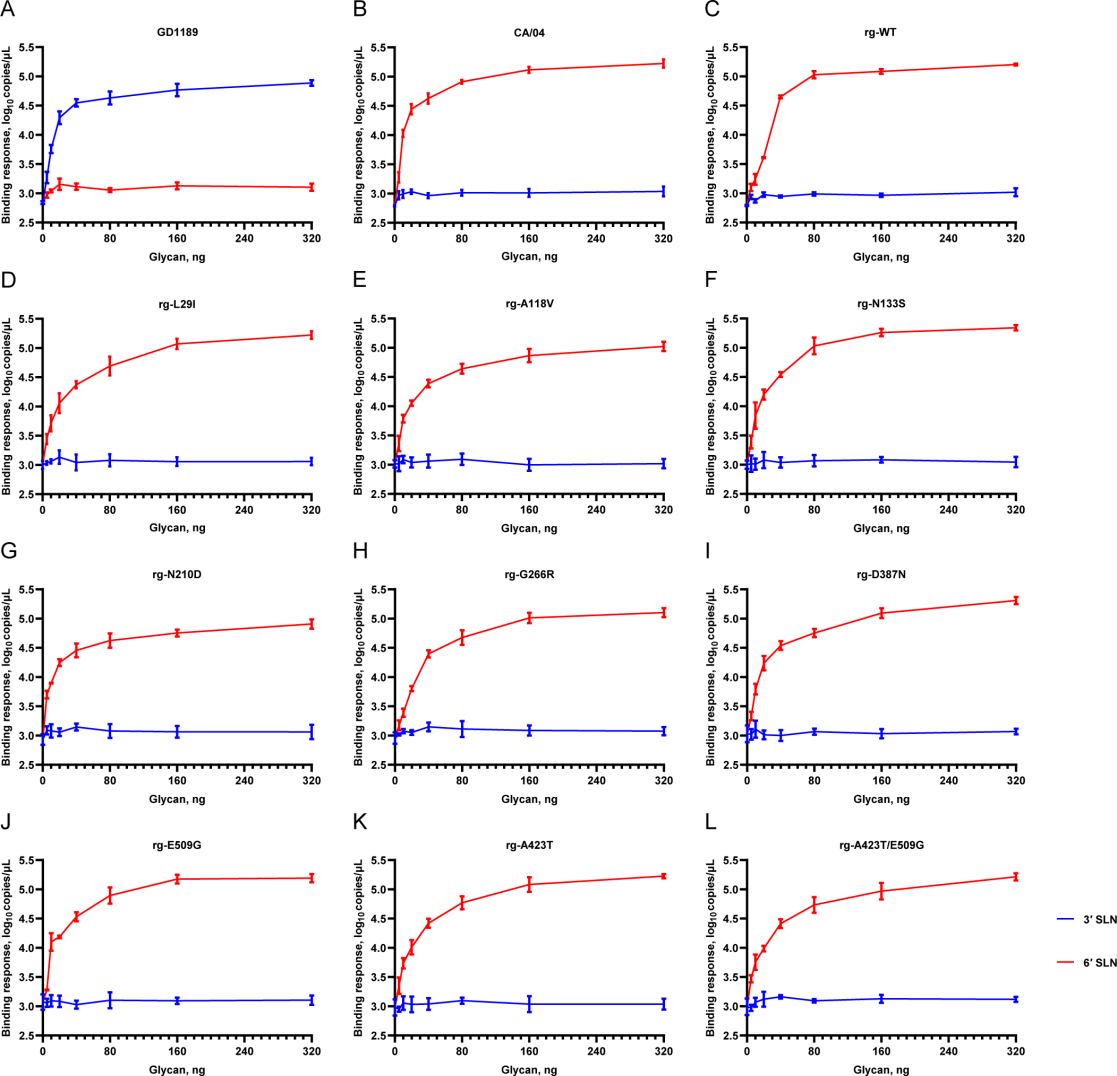
Receptor binding specificity of selected HA-stabilizing H9N2 HA mutant viruses. Receptor-binding properties of the H9N2 HA WT (**C**) and mutants (**D–L**) were evaluated using a solid-phase binding assay to measure their binding to two biotinylated sialylglycopolymers: 3'SLN (avian-like) and 6'SLN (human-like). A/Goose/GuangDong/1189/2023(H5N1)(GD1189, avian type-specific) (**A**) and A/California/04/2009(H1N1) (CA/04, human type-specific) (**B**) viruses were included as controls.

After characterizing the thermostability profiles of the HA mutant viruses, we next evaluated whether the identified HA mutations influenced viral receptor binding specificity using a solid-phase binding assay with biotinylated sialylglycopolymers—3′SLN (avian-type receptor analog) and 6′SLN (human-type receptor analog)—immobilized on streptavidin-coated plates. As controls, the CA/04 (H1N1) virus bound specifically to 6′SLN, while the GD1189 (H5N1) virus bound exclusively to 3′SLN (Fig. 7A and B). Consistent with its human-type receptor preference, the rg-WT (BS19/PR8) virus displayed strong binding to 6′SLN, but negligible binding to 3′SLN (Fig. 7C). Introduction of the thermostability-enhancing HA mutations into the BS19/PR8 background did not alter this receptor preference: all mutant viruses (Fig. 7D through L) maintained strong binding affinity toward 6′SLN, with no detectable increase in binding to 3′SLN. These results indicate that the HA mutations that enhance thermostability do not compromise the human-type receptor binding specificity of the H9N2 virus.

### Effect of HA thermostability mutations on HA activity

**Fig 8:**
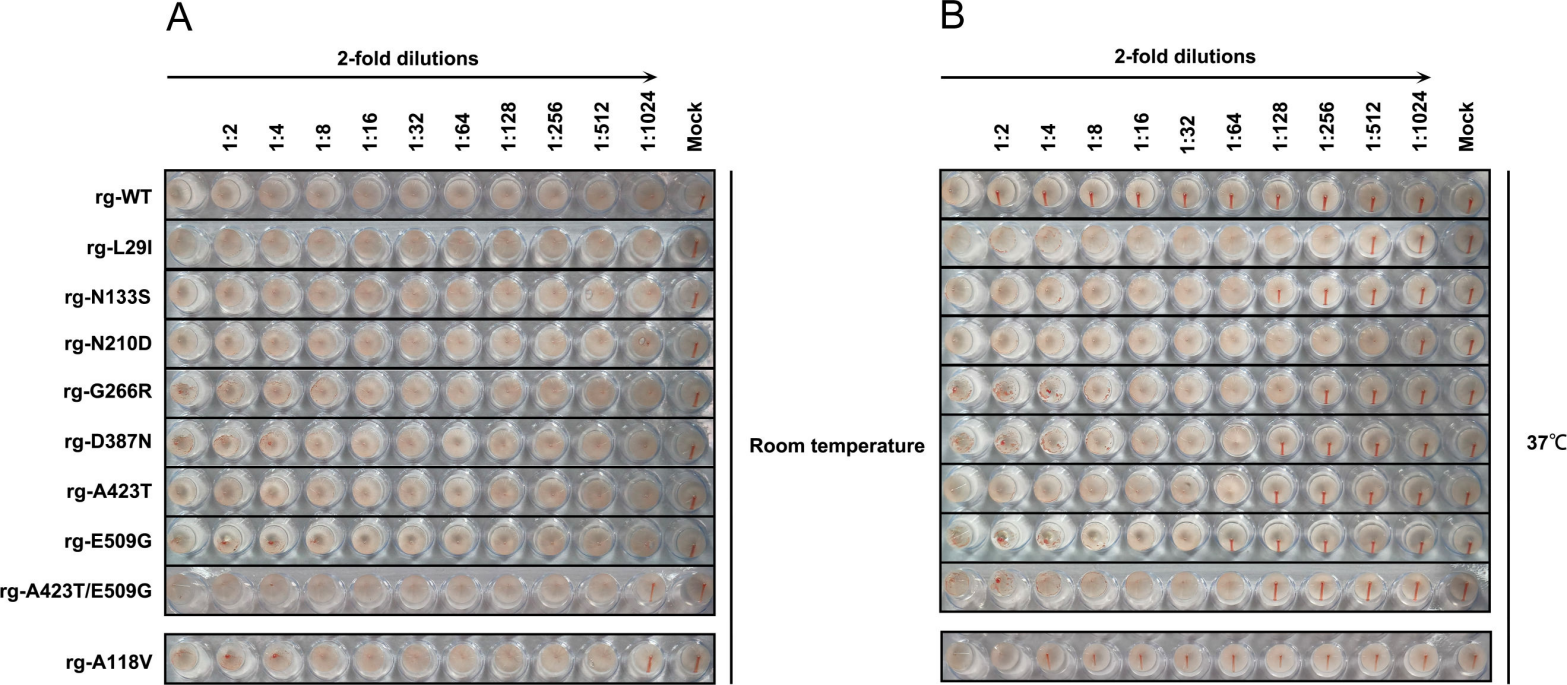
Hemagglutination (HA) activity of HA-stabilizing H9N2 HA mutant viruses. The hemagglutination (HA) activity of mutant viruses was assessed at room temperature (RT) (**A**) and 37°C (**B**) using a standard HA assay with serial 2-fold dilutions of each virus and 0.5% chicken red blood cells (cRBCs).

In our prior study, we observed the emergence of the HA N141K adaptive mutation in the BS19 virus after serial passage in SPF eggs (41). This mutation restored HA activity at 37°C and improved viral thermostability. We therefore sought to determine whether the HA thermostability mutations identified in this study similarly influence HA activity of H9N2 viruses at 37°C. To this end, we performed HA assays using 0.5% cRBCs at both RT (Fig. 8A) and 37°C (Fig. 8B).

As reported previously, the rg-WT exhibited robust HA activity at RT, reaching a titer of 1,024 HA units/50 µL. However, HA activity was abolished at 37°C. In contrast, viruses bearing thermostable HA mutations retained the capacity to effectively agglutinate cRBCs at both RT and 37°C. Although HA titers were generally reduced at 37°C, the mutant viruses maintained demonstrable hemagglutination activity.

### Effect of HA thermostability mutations on viral replication kinetics

**Fig 9:**
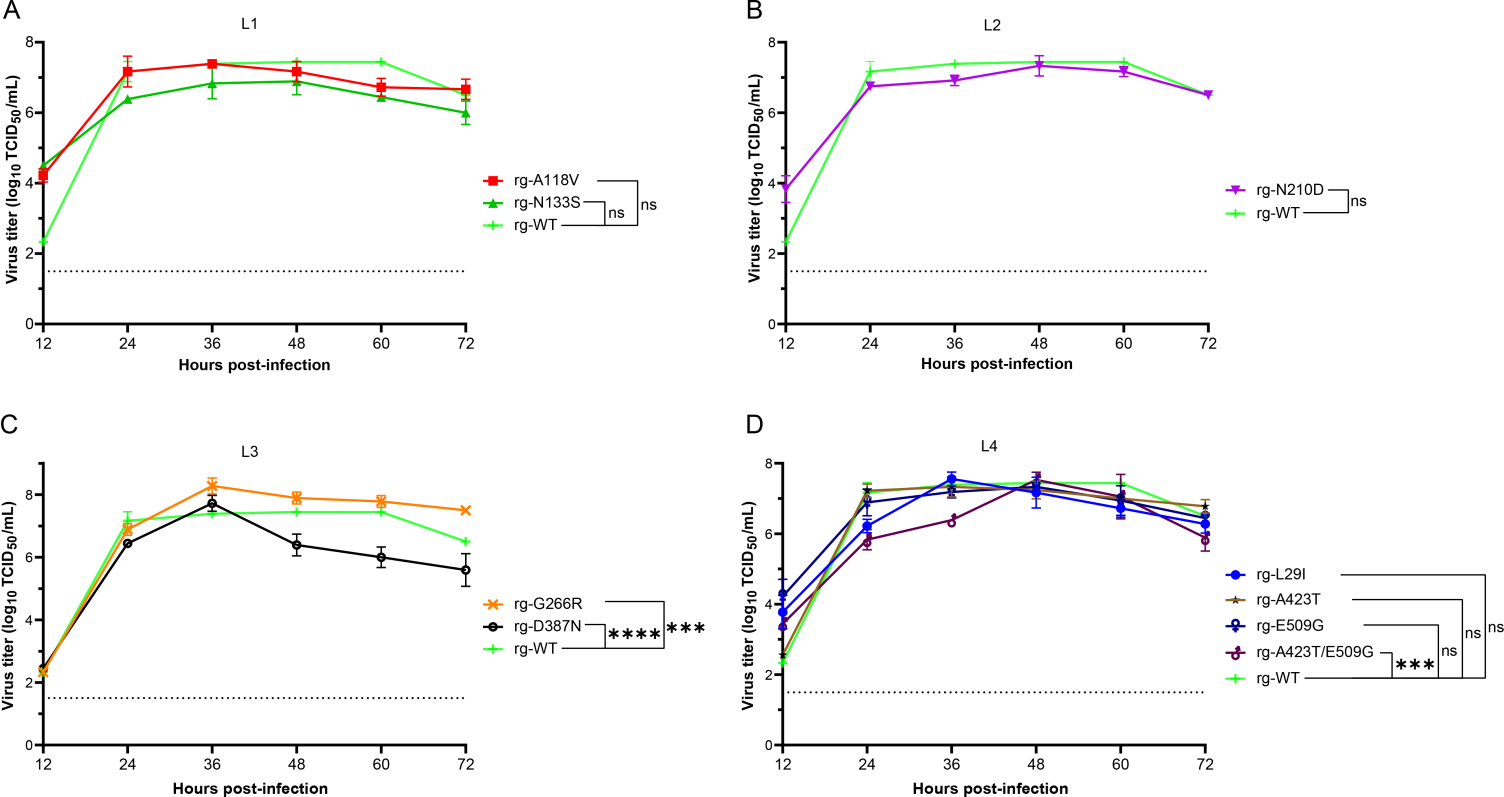
Replication kinetics of wild-type and mutant H9N2 viruses in MDCK cells. The replication capacities of nine HA mutants, identified from each mutant virus library L1 (**A**), L2 (**B**), L3 (**C**), and L4 (**D**), were compared to the wild-type (WT) virus. Multistep growth curves were determined by infecting MDCK cells and titrating the virus titer at the indicated time points. The histogram displays the mean virus titer across the entire replication cycle. Error bars represent the standard deviation (SD) from three independent replicates. Statistical significance was determined by two-way ANOVA, followed by Dunnett’s multiple comparison test (*P* < 0.001, ***P < 0.0001; ns, not significant). The dashed line indicates the limit of detection (LOD) of the titration assay, which was 10^1.5^ TCID_50_/mL.

To assess whether the HA mutations that enhance thermostability impact viral replication efficiency, we compared the multi-step growth kinetics of rg-WT and mutant H9N2 viruses in MDCK cells. As shown in Fig. 9, the mutants exhibited a spectrum of replication phenotypes. The viruses carrying mutations A118V (from library 1), N133S (library 1, Fig. 9A), N210D (library 2, Fig. 9B), L29I (library 3), A423T (library 3), and E509G (library 3, Fig. 9D) demonstrated replication kinetics and peak titers comparable to those of the rg-WT virus. This suggests that the increased HA thermostability in these mutants does not impair viral fitness under standard cell culture conditions. In contrast, the D387N mutant and the A423T/E509G double mutant showed attenuated replication profiles, achieving significantly lower peak titers than the rg-WT virus (*P* < 0.01), which indicates a potential fitness cost associated with these specific mutations (Fig. 9C). Conversely, the G266R mutant replicated to a peak titer that was significantly higher than that of the rg-WT after 36 h post-infection (*P* < 0.001), suggesting that this mutation may confer a replication advantage in addition to its stabilizing effect on the HA protein.

### Impact of thermostability mutations on HA elution kinetics

To investigate the effect of HA protein mutations identified from a thermostability library on the receptor-binding stability of H9N2 virus, we performed erythrocyte elution assays on rg-WT, L29I, A118V, N133S, N210D, G266R, D387N, A423T, E509G, and A423T/E509G mutant viruses. The assay measures the percentage of virus that remains bound to erythrocytes over time at room temperature, reflecting the balance between HA-receptor binding affinity and NA-receptor destroying activity. This provides a functional readout of how thermostability mutations may alter the structural flexibility or conformational dynamics of HA, thereby affecting its dissociation from receptors.

**Fig 10:**
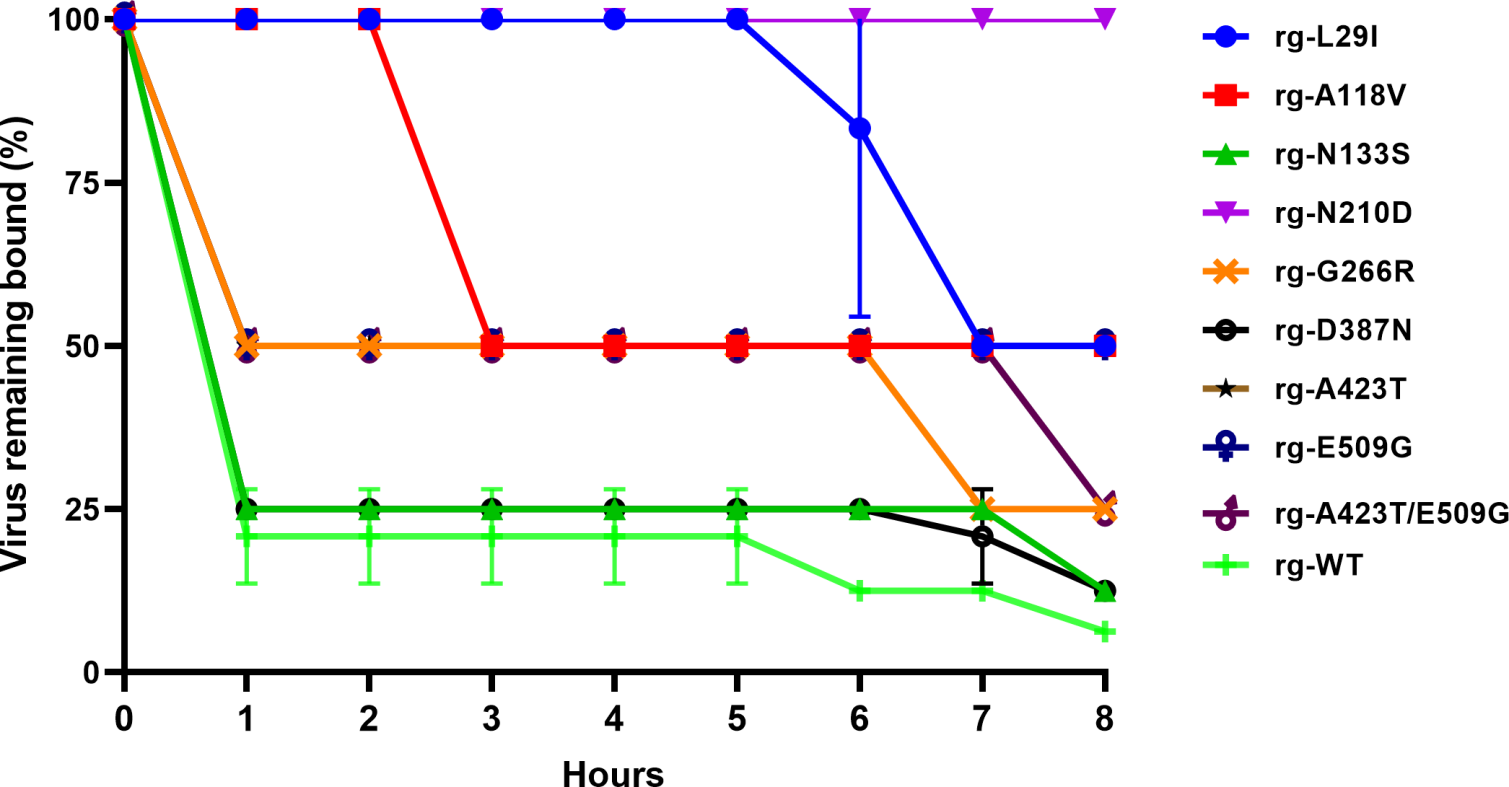
Hemagglutination-elution efficiency of HA-stabilizing H9N2 HA mutant viruses. Wild-type (rg-WT) and mutant viruses were assessed for their hemagglutination-elution efficiency. Viruses were adsorbed to 0.5% chicken red blood cells (cRBCs) at 4°C for 30 min. Elution of viruses from cRBCs was then monitored at room temperature for 8 h. The elution rate is expressed as the percentage of virus eluted over time. Data represent the mean from three independent experiments.

Substantial differences in viral elution kinetics were observed among the mutant viruses compared to rg-WT (Fig. 10). The rg-WT virus displayed rapid elution characteristics, with approximately 75% of viruses eluted within 1 h (25% remaining bound). Several mutants (rg-N133S, D387N, A423T, E509G, and A423T/E509G) showed elution profiles similar to rg-WT. In contrast, viruses with mutations L29I, A118V, and N210D exhibited significantly impaired elution efficiency (*P* < 0.001). The rg-A118V mutant reached only 50% elution after 3 h, while rg-L29I showed a gradual reduction in binding over 7 h. Most notably, rg-N210D maintained stable binding with minimal elution over the 8 h period, demonstrating substantially reduced elution efficiency compared to rg-WT. These findings suggest that specific mutations (L29I, A118V, and N210D) enhance HA-receptor binding stability by potentially increasing the structural rigidity of HA or reducing its dissociation rate from sialic acid receptors.

### Impact of thermostability mutations on acid stability

**Fig 11:**
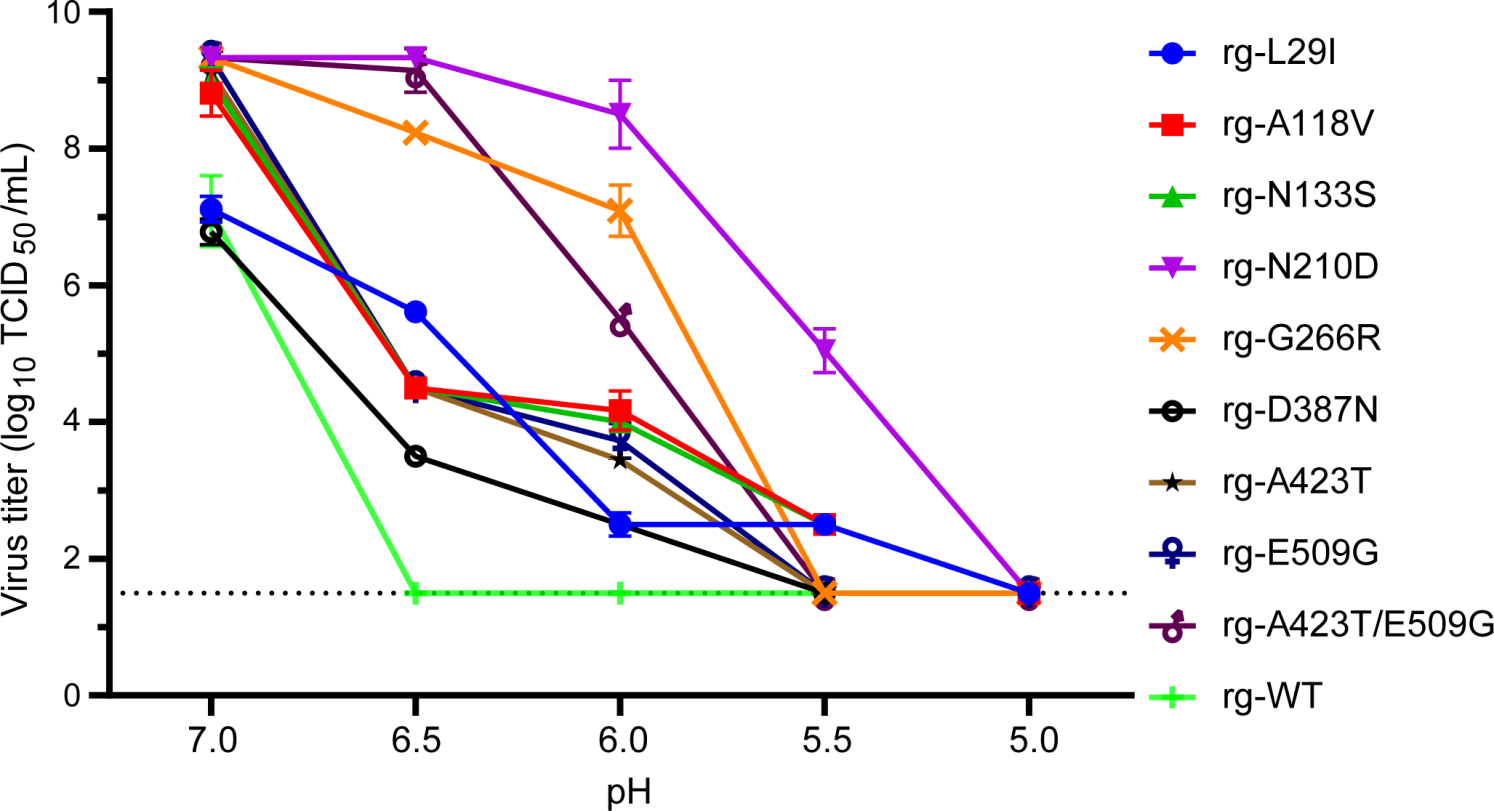
Acid stability profiles of HA-stabilizing H9N2 HA mutant viruses. Acid stability was evaluated for wild-type (rg-WT) and nine mutant viruses (L29I, A118V, N133S, N210D, G266R, D387N, A423T, E509G, and A423T/E509G; all at 106 TCID_50_/100 μL) by measuring viral titers on MDCK cells following incubation at a range of pH values (7.0, 6.5, 6.0, 5.5, 5.0, and 4.5). Data represent the mean from three independent experiments. The dashed line indicates the limit of detection (LOD) of the titration assay, which was 10^1.5^ TCID_50_/mL.

Compared to the rg-WT virus, the L29I, A118V, N133S, N210D, G266R, D387N, A423T, E509G, and A423T/E509G mutant viruses exhibited increased acid stability (Fig. 11), suggesting that these thermostabilizing mutations may also influence the HA protein’s response to low-pH environments encountered during viral entry. Specifically, rg-WT, D387N, A423T, A118V, N133S, and E509G displayed a rapid decline in activity at pH 6.5. The rg-WT virus titer decreased by 7.50 log10 units and lost HA activity, while L29I, D387N, A423T, A118V, N133S, and E509G maintained HA activity (≥3.50 log_10_ units), with titers gradually decreasing at lower pH, indicating greater resistance to acid-induced inactivation. D387N, A423T, and E509G completely lost the ability to agglutinate CRBCs at pH 5.5, whereas L29I retained activity until pH 5.0, demonstrating a slightly more robust acid-stable phenotype. N210D, G266R, and A423T/E509G showed relatively consistent viral titers at pH 6.5. Upon decreasing the pH to 6.0, N210D and G266R maintained high viral titers, while A423T/E509G exhibited a 3.80 log10 unit reduction in viral activity. G266R and A423T/E509G lost HA activity at pH 5.5, whereas N210D retained a viral titer of 5.00 log_10_ units, maintaining activity until pH 5.0, further emphasizing the superior acid stability conferred by the N210D mutation. In summary, the N210D, G266R, L29I, A118V, and N133S mutations enhanced viral acid stability, particularly the N210D mutation, which conferred a strong acid-stable phenotype upon its introduction into rg-WT.

## DISCUSSION

The contribution of low HA stability to the biology of H9N2 AIV remains a key area of investigation. To identify the key residues affecting the HA thermostability of H9N2 AIV, we used an integrated strategy combining error-prone PCR, site-directed mutagenesis, and reverse genetics to create and characterize a diverse library of H9N2 HA variants. This approach identified a panel of mutations, including the single amino acid substitutions L29I, N133S, N210D, G266R, and D387N, and the double mutant A423T/E509G, that enhanced HA thermostability at elevated temperatures. Our analysis of H9N2 HA variants harboring thermostabilizing mutations has revealed a complex interplay between HA stability and acid tolerance (Table 1).

**TABLE 1:** Effects of amino acid mutations on the thermal stability and fusion activity of virus strains^*a*^

Virus	TCID_50_ titer after 48°C, 1 h (log_10_ TCID_50_/μL)	HA titer after 48°C, 1 h (log_2_)	Critical pH (stability)	Glycan receptor binding
rg-WT	0	0	6.5	α-2,6
rg-A118V	0	0	5	α-2,6
rg-L29I	3.28	1.67	5	α-2,6
rg-N133S	4.24	3	5	α-2,6
rg-N210D	6.28	7	5	α-2,6
rg-G266R	5.17	5.33	5.5	α-2,6
rg-D387N	4.03	3	5.5	α-2,6
rg-A423T	2.44	0.33	5.5	α-2,6
rg-E509G	2.33	0	5.5	α-2,6
rg-A423T/E509G	5.39	4.67	5.5	α-2,6

^*a*^ The H9N2 mutant viruses were constructed by reverse genetics, with internal genes derived from the PR8 virus. Data shown are representative of at least three independent experiments.

Analysis of the spatial distribution of identified mutations revealed that N133S (L1) and N210D (L2) reside within the receptor-binding domain of HA, while G266R (L3), D387N (L3), A423T (L4), and E509G (L4) are located in the HA stem region. Notably, the adaptive mutation L29I is also situated within the stem domain. These findings indicate that HA thermostability is modulated by residues in both the globular head and stem regions of the protein, suggesting a complex interplay of structural elements governing thermal stability. Consistent with this notion, previous studies of H5N1 HA have identified conserved mechanisms of thermostabilization across different influenza subtypes (46). For example, the I29M mutation in H5N1 HA enhances thermostability, mirroring our observation that the analogous L29I mutation in H9N2 HA confers a similar benefit. Furthermore, the K387I substitution in H5N1 HA lowers the pH threshold for membrane fusion and improves thermostability, resulting in increased viral replication and virulence in mice (48, 49). Interestingly, we found that the D387N mutation in H9N2 HA also augmented thermostability, suggesting a parallel mechanism of action.

Since 2013, H9N2 viruses circulating in China have shown a clear trend toward increased affinity for human-type receptors (50, 51). This adaptation is driven by a combination of HA mutations that broaden receptor tropism (e.g., Q227M, D145G, S119R, and R246K) and others that specifically enhance human-type receptor binding (e.g., A160N, Q156R, and T205A) (51). Furthermore, the I155T and G192R mutations in HA promote viral adaptation in mammalian models, such as mice and ferrets, by increasing affinity for human-type receptors (10, 52). HA mutations N158D, T163I, D193N, and N231D are also associated with enhanced H9N2 virus binding to human-type receptors (53–56).

Our study’s focus on the BS19 HA is particularly relevant, given that it has accumulated several of these human-adaptive mutations, resulting in a marked preference for human-type receptors. Crucially, our thermostable HA mutants retained preferential binding to α-2,6 sialic acid receptors without a concomitant increase in binding to α-2,3 sialic acid receptors. This finding suggests that enhanced thermostability can be achieved without compromising human receptor specificity. It is well-established that effective airborne transmission of H5 viruses in ferrets necessitates a combination of factors, including a lower pH activation threshold, increased HA thermostability, and a shift in HA receptor-binding preference from avian to human receptors (15, 57). Our observation that H9N2 HA thermostable mutants maintain binding to α-2,6 receptors and exhibit improved thermostability provides new insights into the evolutionary trajectory and transmission potential of these viruses. Consistent with this, viruses harboring HA thermostability mutations retained hemagglutination activity at 37°C, suggesting that HA thermostability mutations, such as N141K, can preserve virus binding to CRBCs at elevated temperatures.

Prior research on H5 influenza viruses has demonstrated that while mutations enabling human-type receptor binding can compromise HA stability, compensatory mutations acquired during adaptation are crucial to restore stability (46). These stabilizing mutations concomitantly lower the pH threshold required for HA conformational change and membrane fusion in acidic endosomes. By promoting HA-mediated fusion at a higher pH, such mutations facilitate viral entry and subsequent spread. Given the structural and functional conservation of HA across influenza subtypes, it is plausible that the thermostability mutations identified in H9N2 HA operate through analogous mechanisms. If confirmed, this would highlight the significance of HA thermostability in viral adaptation to mammalian hosts and offer a valuable framework for elucidating the molecular basis of H9N2 adaptation and transmission. Our findings indicate that specific mutations (L29I, A118V, and N210D) strengthen HA-receptor binding stability, likely by increasing the structural rigidity of HA or reducing its dissociation rate from sialic acid receptors. The observed delay in elution may stem from mutations that stabilize the HA trimer, thereby necessitating greater neuraminidase (NA) activity to achieve elution rates comparable to the WT. These insights reveal how thermostability mutations can influence viral receptor-binding properties beyond structural stabilization alone.

To assess the prevalence of the identified HA thermostability mutations in naturally circulating H9N2 viruses, we queried the GISAID EpiFlu database. Among human H9N2 isolates, we detected the N210D substitution in eight isolates and the G266R substitution in four isolates, with N210D appearing to be more prevalent in recent isolates. We also identified the N210D substitution in a single ferret H9N2 isolate, and the A423T substitution in a single swine H9N2 isolate; however, the combined A423T/E509G mutations were not observed in any analyzed sequences. Notably, these sites appear to be highly conserved in avian H9N2 strains, suggesting that these specific mutations may not be under strong positive selection in avian reservoirs. Nevertheless, the apparent increase in the prevalence of N210D in recent human H9N2 isolates suggests a potential selective advantage in the human host, warranting further investigation. Future studies are needed to fully elucidate the selective pressures driving the emergence and maintenance of these mutations in natural H9N2 populations and to dissect their potential role in host-specific adaptation.

In conclusion, this study identified key mutations that enhance HA thermostability in H9N2 AIV, offering a foundation for further investigations into the molecular mechanisms governing H9N2 transmission. While stable HA proteins may offer advantages in vaccine production, storage, or immunogenicity, future research should focus on exploring the feasibility of developing improved thermostable influenza vaccines based on these findings.

## Data Availability

All data generated or analyzed during this study are contained within this article. The corresponding raw data supporting the findings are available from the corresponding author upon reasonable request.
